# 4CMenB Breadth of Immune Response, Immunogenicity, and Safety: Results From a Phase 3 Randomized, Controlled, Observer Blind Study in Adolescents and Young Adults

**DOI:** 10.1093/ofid/ofae638

**Published:** 2024-10-30

**Authors:** Terry Nolan, Chiranjiwi Bhusal, Jiří Beran, Mark Bloch, Benhur S Cetin, Ener C Dinleyici, Daniel Dražan, Satu Kokko, Susanna Koski, Outi Laajalahti, Joanne M Langley, Mika Rämet, Peter C Richmond, Peter Silas, Bruce Tapiero, Florence Tiong, Mary Tipton, Benita Ukkonen, Betul Ulukol, Maria Lattanzi, Mauro Trapani, Arnold Willemsen, Daniela Toneatto, the QUINTET study group, Ronald Ackerman, Renata Adamovska, Eugene Athan, Kwabena Ayesu, Jiří Beran, David Bernard, Chiranjiwi Bhusal, Mark Bloch, William Byars, Robert Carter, Benhur Cetin, Maia Chakerian, Marije Dalebout, Ferdinandus de Looze, Ener Cagri Dinleyici, Marc Dionne, Daniel Dražan, Peter Dzongowski, Rand Farjo, Daniel Finn, George Freeman, Ryan Gottfredson, Paul Grubb, Anil Gupta, Tolga Ince, Robert Jeanfreau, Jake Jones, James Kellner, Kaia Kiiroja, Satu Kokko, Susanna Koski, Joanne Langley, Outi Laajalahti, Maria Lattanzi, Isabelle Lechevin, Hemalini Mehta, Sandra Meisalu, Danielle Morelle, Terry Nolan, Alexander Osowa, Pauliina Paavola, Minesh Patel, Miroslav Pavlasek, Enrique Pelayo, Mika Rämet, Stefanie Raulier, Peter Richmond, Walter Rok, Rambod Rouhbakhsh, Manish Sadarangani, Yamirka Sanchez, Martin Schear, John Scott, Ilkka Seppä, Peter Silas, William Simon, Martina Spaziererova, Jonathan Staben, Joseph Surber, Bruce Tapiero, Florence Tiong, Mary Tipton, Daniela Toneatto, Mauro Trapani, Benita Ukkonen, Betul Ulukol, Marie-Louise Vachon, Noah Vale, Dominique Wauters, Arnold Willemsen, Josef Zemanek

**Affiliations:** Vaccine and Immunisation Research Group, Peter Doherty Institute at the University of Melbourne, and Murdoch Children's Research Institute, Melbourne, Victoria, Australia; GSK, Amsterdam, The Netherlands; Vaccination and Travel Medicine Centre, Hradec Králové, Czechia; Holdsworth House Medical Practice, Sydney, NSW, Australia; Department of Medicine, Kirby Institute, University of New South Wales, Sydney, NSW, Australia; Department of Pediatric Infectious Diseases, Erciyes University Faculty of Medicine, Kayseri, Türkiye; Eskisehir Osmangazi University Faculty of Medicine, Eskisehir, Türkiye; General Practice for Children and Adolescents, Jindřichův Hradec, Czechia; Oulu Vaccine Research Clinic, FVR Finnish Vaccine Research and Tampere University, Oulu, Finland; Helsinki South Clinic, FVR Finnish Vaccine Research and Tampere University, Finland; FVR Finnish Vaccine Research and Tampere University, Seinäjoen Rokotetutkimusklinikka, Seinäjoki, Finland; Canadian Center for Vaccinology (Dalhousie University, IWK Health and Nova Scotia Health), Halifax, NS, Canada; Faculty of Medicine and Health Technology, FVR Finnish Vaccine Research, Tampere University, Tampere, Finland; University of Western Australia School of Medicine and Vaccine Trials Group, Telethon Kids Institute, Nedlands, Washington, Australia; Wee Care Pediatrics, Syracuse, Utah, USA; CHU Sainte-Justine, Montreal, QC, Canada; Infectious Diseases Division, Department of Pediatrics, University of Montreal, QC, Canada; AusTrials (Wellers Hill), Tarragindi, QLD, Australia; Copperview Medical Center, South Jordan, Utah, USA; FVR Finnish Vaccine Research and Tampere University, Espoo Clinic, Espoo, Finland; Department of Pediatrics, Ankara University School of Medicine, Ankara, Türkiye; GSK, Siena, Italy; GSK, Siena, Italy; GSK, Amsterdam, The Netherlands; GSK, Siena, Italy

**Keywords:** 4CMenB, bactericidal assay, breadth of immune response, meningococcal vaccine, *Neisseria meningitidis*

## Abstract

**Background:**

Meningococcal serogroup B (MenB) strains are highly diverse. Breadth of immune response for the MenB vaccine, 4CMenB, administered at 0–2, 0–6, or 0–2–6 months, was demonstrated by endogenous complement-human serum bactericidal antibody (enc-hSBA) assay against an epidemiologically relevant panel of 110 MenB strains.

**Methods:**

In a phase 3 trial, 3651 healthy 10- to 25-year-old participants were randomized 5:5:9:1 to receive 4CMenB (0–6 schedule), 4CMenB (0–2–6 schedule), investigational MenABCWY vaccine, or control MenACWY-CRM vaccine. The primary objectives were to evaluate safety and demonstrate breadth of immune response by enc-hSBA assay against the MenB strain panel using test-based (percentage of samples without bactericidal activity against strains after 4CMenB vs control vaccination) and responder-based (percentage of participants whose postvaccination sera kill ≥70% strains) approaches. Success was demonstrated with 2-sided 97.5% confidence interval (CI) lower limit >65%. Immunogenicity was assessed by traditional hSBA assay against four indicator strains.

**Results:**

Breadth of immune response (test-based) was 78.7% (97.5% CI, 77.2–80.1), 81.8% (80.4–83.1), 83.2% (81.9–84.4) for the 0–2, 0–6, and 0–2–6 schedules, respectively, and (responder-based) 84.8% (81.8–87.5), 89.8% (87.2–92.0), and 93.4% (91.2–95.2), respectively. No clinically relevant differences in immunogenicity were observed across schedules. 4CMenB was well tolerated.

**Conclusions:**

The 2-dose (0–2, 0–6) 4CMenB schedules met predefined criteria for success for both breadth of immune response endpoints against a diverse MenB strain panel, had comparable immunogenicity, and safety in line with the established 4CMenB safety profile. The 3-dose schedule provided no additional immunological benefit, supporting use of the 4CMenB 0–2 schedule.

## Teaser

- 4CMenB 2- and 3-dose schedules met predefined success criteria for breadth of immune response endpoints, assessed by endogenous complement-hSBA assay against a panel of 110 diverse MenB strains
- Breadth of immune response in adolescents and young adults was consistent with real-life vaccine effectiveness

## Graphical Abstract

**Graphical Abstract:**
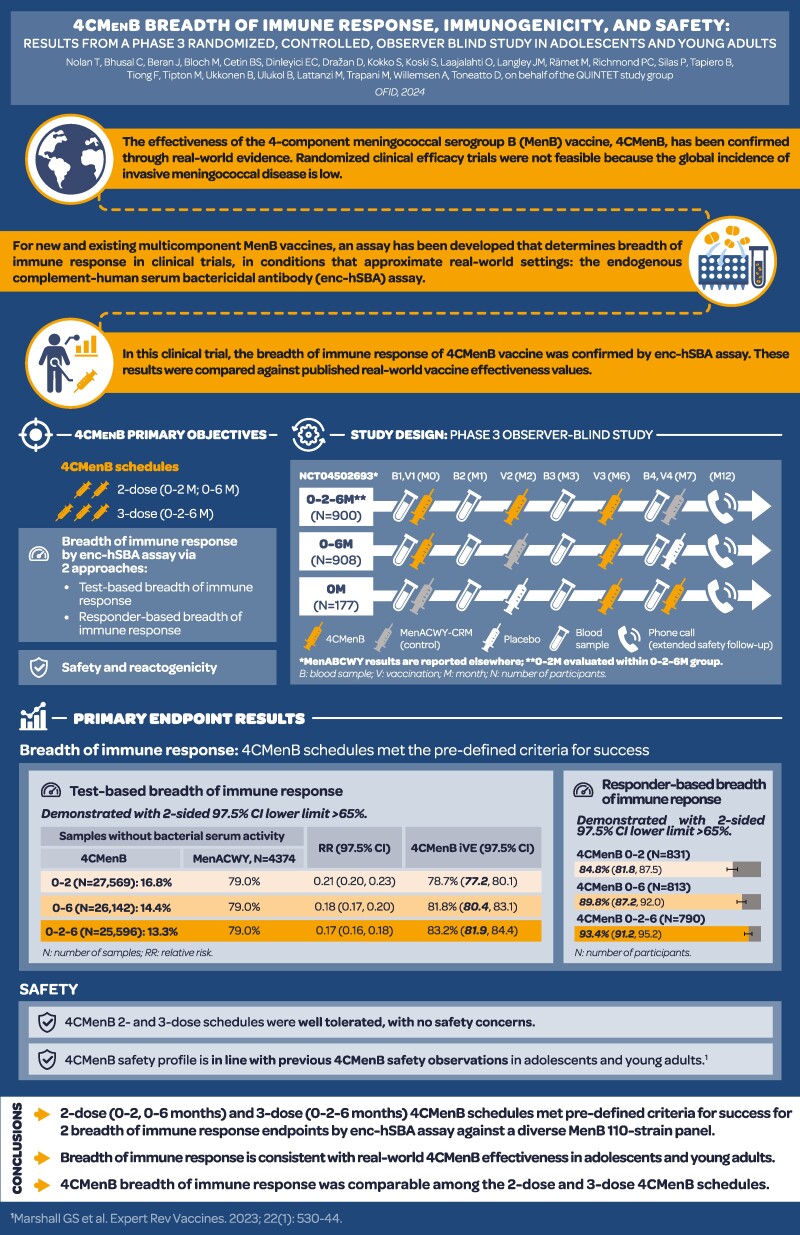
A graphical abstract is available online.

Invasive meningococcal disease (IMD), caused by *Neisseria meningitidis* and primarily manifesting as meningitis or septicemia [1], progresses rapidly, is life-threatening, and can have severe long-term consequences for survivors [2–4]. Six *N meningitidis* serogroups (A, B, C, W, X, Y) cause most cases of IMD [5]. Although effective vaccines are available against meningococcal serogroups ACWY based on capsular polysaccharides [6, 7], the meningococcal serogroup B (MenB) capsular polysaccharide has poor immunogenicity [8], leading to the development of 2 protein-based MenB vaccines: the 4-component 4CMenB vaccine (Bexsero, GSK) [9] and the bivalent MenB-FHbp vaccine (Trumenba, Pfizer), which includes 1 recombinant factor H binding protein (fHbp) variant from each subfamily [10]. 4CMenB includes 3 recombinant protein antigens, *Neisseria* adhesin A (peptide 3.8), neisserial heparin-binding antigen (peptide 2), and fHbp (peptide 1.1, B24 variant), and detergent-extracted outer membrane vesicles from a New Zealand outbreak strain, containing Porin A (serosubtype P1.4) as the immunodominant antigen [9, 11].

Because conventional efficacy studies of vaccines against low-incidence diseases such as IMD require unfeasibly large numbers of participants, initial approval of both MenB vaccines relied on the accepted surrogate of protection: immunogenicity results against vaccine antigen-specific MenB indicator strains, generated by the human serum bactericidal antibody (hSBA) assay using complement sourced exogenously [12]. 4CMenB was licensed in the European Union in 2013 and gained accelerated approval in the United States (US) in 2015, with a postmarketing commitment to confirm breadth of immune response in a clinical trial [13], and is currently registered in 55 countries.

Large-scale, real-world studies in the United Kingdom, Italy, Portugal, Spain, and Australia [14–23] and disease outbreak control programs in Canada and the US [24–26] have confirmed the impact and effectiveness of 4CMenB against MenB disease when administered in various dose schedules to different age groups. Real-world evidence is the only means of confirming MenB vaccine effectiveness because MenB protein antigens are highly diverse in genetic features and expression levels [8, 27, 28]. Consequently, *in vitro* methods are needed to assess the performance of MenB vaccines in randomized clinical trials before vaccine licensure and before real-world evidence is generated.

The traditional hSBA assay is useful for measuring MenB vaccine immunogenicity but large serum volumes are required to test large numbers of diverse circulating strains and an appropriate source of exogenous complement without antibodies is needed for each test strain [8]. The endogenous complement-hSBA (enc-hSBA) assay uses instead endogenous complement present in each vaccinated person's serum [29]. This assay thereby accounts for intersubject variability in complement-mediated bactericidal killing of MenB strains and the synergistic killing effect of antibody responses elicited by all vaccine antigens combined [30], rather than assessing single antigen recognition as the basis for bacterial killing, as in the traditional hSBA assay. The enc-hSBA assay has been validated against a panel of 110 MenB strains randomly selected from 442 strains collected between 2000 and 2008 in the US by the Centers for Disease Control and Prevention [31, 32]. The 442-strain panel appears to be stable over time [33], and extensive characterization shows the repertoire of antigen genotypes in the 110 MenB strain panel represents approximately 95% of invasive MenB strains circulating in the US and overall 89% of invasive strains collected in the US, Canada, Europe, and Australia [34]. Hence, enc-hSBA assay of this epidemiologically relevant panel enables evaluation of breadth of immune response, the vaccine's ability to induce a bactericidal immune response against a broad panel of MenB strains in randomized clinical trials, in conditions that are close to real-world settings [29, 35].

We conducted a multinational phase 3 study to demonstrate breadth of immune response, immunogenicity, and safety for 4CMenB and an investigational pentavalent MenABCWY vaccine when administered to healthy adolescents and young adults. In this study, we determine for the first time 4CMenB breadth of immune response when administered in a 2-dose or 3-dose schedule via enc-hSBA assay of the 110 MenB strain panel. We also report results on 4CMenB immunogenicity by traditional hSBA assay, together with safety and reactogenicity outcomes. Results for the investigational MenABCWY vaccine will be reported separately.

## METHODS

### Study Design and Participants

This phase 3, randomized, controlled, observer-blind study (NCT04502693) was conducted between August 2020 and September 2022 at 114 centers in 7 countries: US (65 centers), Canada and Czechia (12 centers each), Finland (10 centers), Australia (7 centers), Türkiye (5 centers), and Estonia (3 centers). A summary of the study design is available at www.gsk-studyregister.com/en/ (study identifier: 205416) and study endpoints are listed at ClinicalTrials.gov (www.clinicaltrials.gov/study/NCT04502693). The study was conducted in accordance with the Declaration of Helsinki and Good Clinical Practice and approved by the appropriate ethics committees. Protocol amendments resulting from the COVID-19 pandemic allowed for widened visit windows, COVID-19 vaccination, home visits if required, and data collection on COVID-19 symptoms and treatment. Written informed consent and, for children, assent was provided by participants or their parents or legally acceptable representatives.

Healthy individuals aged 10 to 25 years were recruited, including those who had no history of meningococcal disease and had not been vaccinated with a MenB vaccine. A single MenACWY dose received ≥4 years previously and MenC vaccination (last dose received at age ≤24 months) were permitted. Abnormal function or modification of the immune system was an exclusion criterion. All inclusion and exclusion criteria for participation are listed in the study protocol (www.gsk-studyregister.com/en/trial-details/?id=205416#documents-section).

**Figure 1.**
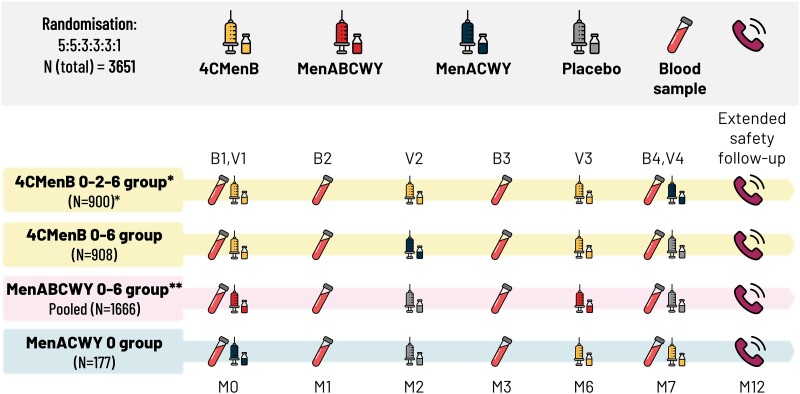
Study design, with numbers of participants randomized to each group. *4CMenB 0–2–6 M and 0–2 M schedules evaluated. **Three MenABCWY groups, each receiving 1 of 3 lots of MenACWY component of MenABCWY vaccine. 4CMenB 0–2–6 group, 3 doses of 4-component meningococcal serogroup B (4CMenB) vaccine at study months 0, 2, 6; 4CMenB 0–6 group, 2 doses of 4CMenB at study months 0, 6; MenABCWY group, 2 doses of meningococcal serogroups ABCWY vaccine at months 0, 6; MenACWY group, meningococcal serogroups ACWY-CRM glycoconjugate vaccine at month 0; B, blood sample; M, study month; N, number of participants; V, study visit.

Participants were randomized (5:5:3:3:3:1 ratio) to 1 of 6 parallel groups (Figure 1): the 4CMenB 0–2–6 group received 4CMenB at study months 0, 2, and 6; the 4CMenB 0–6 group received 4CMenB at 0 and 6 months; 3 MenABCWY groups received the investigational MenABCWY vaccine (each group receiving 1 of 3 MenACWY CRM_197_ conjugate vaccine, MenACWY-CRM, component production lots) at 0 and 6 months; the MenACWY group (control) received MenACWY-CRM (Menveo, GSK) at month 0. Additional doses of MenACWY-CRM (4CMenB groups) or 4CMenB (MenACWY group) (Figure 1) were administered in line with the standard of care.

Allocation to study group at each site was conducted via a central randomization system using a minimization procedure accounting for region (US/non-US countries), previous MenACWY vaccination (yes/no), and age category (10–17/18–25 years of age). There were 6 study visits and regular safety follow-ups through telephone calls to each participant, with the final telephone call 12 months after the first vaccine dose (Figure 1).

The study was observer blinded (ie, participants and those responsible for evaluating study endpoints were unaware of group allocation) and vaccine and placebo injections were prepared and administered by unblinded trial staff who did not participate in the clinical evaluations or assays. Enc-hSBA testing was done by laboratory personnel blinded for group allocation, visit, and participant identification.

For 4CMenB, primary study objectives were to demonstrate breadth of immune response by enc-hSBA assay against a panel of 110 MenB strains, using test-based and responder-based approaches, as described in the statistical analysis section. The 4CMenB 0–2 schedule was evaluated within the 4CMenB 0–2–6 group. The safety and reactogenicity of 4CMenB was assessed as a primary objective, and immunogenicity was assessed by traditional hSBA assay as a secondary study objective.

4CMenB and MenACWY-CRM vaccine compositions were described previously [36, 37]. The placebo was a 0.9% saline solution. Vaccines were prepared as 0.5 mL doses and administered intramuscularly into the deltoid region, preferably of the nondominant arm.

### Serological Analyses

Details of the enc-hSBA assay [29, 35] and 110 MenB strain panel [34, 35] were published previously. Blood samples were taken at study months 0, 1, 3, and 7 (Figure 1). Serum samples for the enc-hSBA assay underwent a procedure to preserve endogenous complement activity [29]. It was not technically feasible to test each serum sample from each vaccinated participant against all 110 MenB test strains. Therefore, for each sample, 35 strains were randomly selected by the randomization officer for testing; each sample had up to 35 valid test results. Each sample was diluted 1:4 before testing. The enc-hSBA assay measures the capability of sera from immunized individuals to elicit bactericidal killing of MenB strains at 1:4 dilution; hSBA titer ≥1:4 is the generally accepted surrogate measure of protection against IMD [8]. For each tested strain, the result is considered positive if ≥50% of incubated bacteria are killed.

4CMenB immunogenicity was assessed by hSBA assay against 4 indicator strains, each expressing 1 of the 4 vaccine antigens on its surface [36]. Immunogenicity endpoints included percentage of participants with a 4-fold rise in hSBA titer and percentage with hSBA titer ≥ lower limit of quantitation (LLOQ), and hSBA geometric mean titer (GMT). Four-fold rise in hSBA titer, LLOQ, and hSBA assay limits are defined in the Supplement. Minor changes to the limits requested by the Center for Biologics Evaluation and Research (US Food and Drug Administration) had no clinically relevant impact on the results.

### Safety Analyses

Participants were observed for at least 30 minutes after each vaccine dose for immediate adverse events (AEs). Solicited local (pain, erythema, swelling, induration at the injection site) and systemic (fatigue, nausea, myalgia, arthralgia, headache, fever) AEs were reported by participants on electronic diaries for 7 days (including day of vaccination) following each vaccine dose. The severity of solicited AEs was classified as mild, moderate, or severe, with severe defined as preventing normal activity, apart from severe erythema, swelling, and induration, which was defined as diameter >100 mm. Fever was defined as body temperature ≥38.0 °C and severe fever as body temperature ≥40 °C.

The percentages of participants with unsolicited AEs up to 30 days after each dose, and with serious AEs (SAEs), medically attended AEs, AEs leading to withdrawal, and AEs of special interest (including arthritis and potential immune-mediated diseases or disorders [38]) in the 12-month trial period were assessed. The causal relationship of unsolicited AEs to vaccination was assessed by trial investigators.

### Statistical Analyses

Estimating a possible dropout rate of 25%, enrollment of 3651 participants (including 912 in each 4CMenB group) was planned to give an evaluable result providing 90% power to reject all hypotheses for the 4CMenB primary objectives. Test-based and responder-based approaches were used to demonstrate breadth of immune response. Based on results from a phase 2 study [35], 684 evaluable participants per 4CMenB group was estimated to have ≥99.9% power to demonstrate breadth of immune response by test-based analysis and ≥97% power by responder-based analysis.

The test-based endpoint was analyzed in the per-protocol set (participants who received at least one study dose and had post-vaccination effectiveness or immunogenicity data and no major protocol deviations). This was computed as 100% × (1 – relative risk), with relative risk defined as the percentage of samples without bactericidal serum at 1:4 dilution in the 4CMenB group divided by the percentage in the control MenACWY group. Test-based breadth of immune response was analyzed using a generalized linear model, with vaccine group, strain, and randomization factors as independent variables (ie, [as defined previously] region, age category, and previous MenACWY vaccination) and used with binary distribution and link function log to compute the log relative risk and the corresponding 97.5% confidence interval (CI). This CI was used because alpha = 0.05 was split equally between 4CMenB and MenABCWY vaccine objectives (alpha = 0.025; 97.5% CI each). Responder-based breadth of immune response was measured as the percentage of participants in the full analysis set (participants who received at least 1 study dose and had postvaccination effectiveness or immunogenicity data) whose sera killed ≥70% tested strains (by enc-hSBA assay) at 1 month after the second or third 4CMenB dose. For both breadth of immune response endpoints, success was demonstrated if the lower limit of 2-sided 97.5% CI was >65%.

The secondary immunogenicity objectives, hSBA GMT, percentage of participants with 4-fold rise in hSBA titer, and percentage with hSBA titer ≥ LLOQ were analyzed for each MenB indicator strain, with 2-sided 95% Clopper-Pearson CIs [39]. All participants who received study vaccination and provided safety data were included in the descriptive safety analyses.

Statistical analyses were performed using Statistical Analysis System Life Science Analytics Framework with SAS version 9.4 (SAS Institute Inc., US).

## RESULTS

### Participants

**Figure 2.**
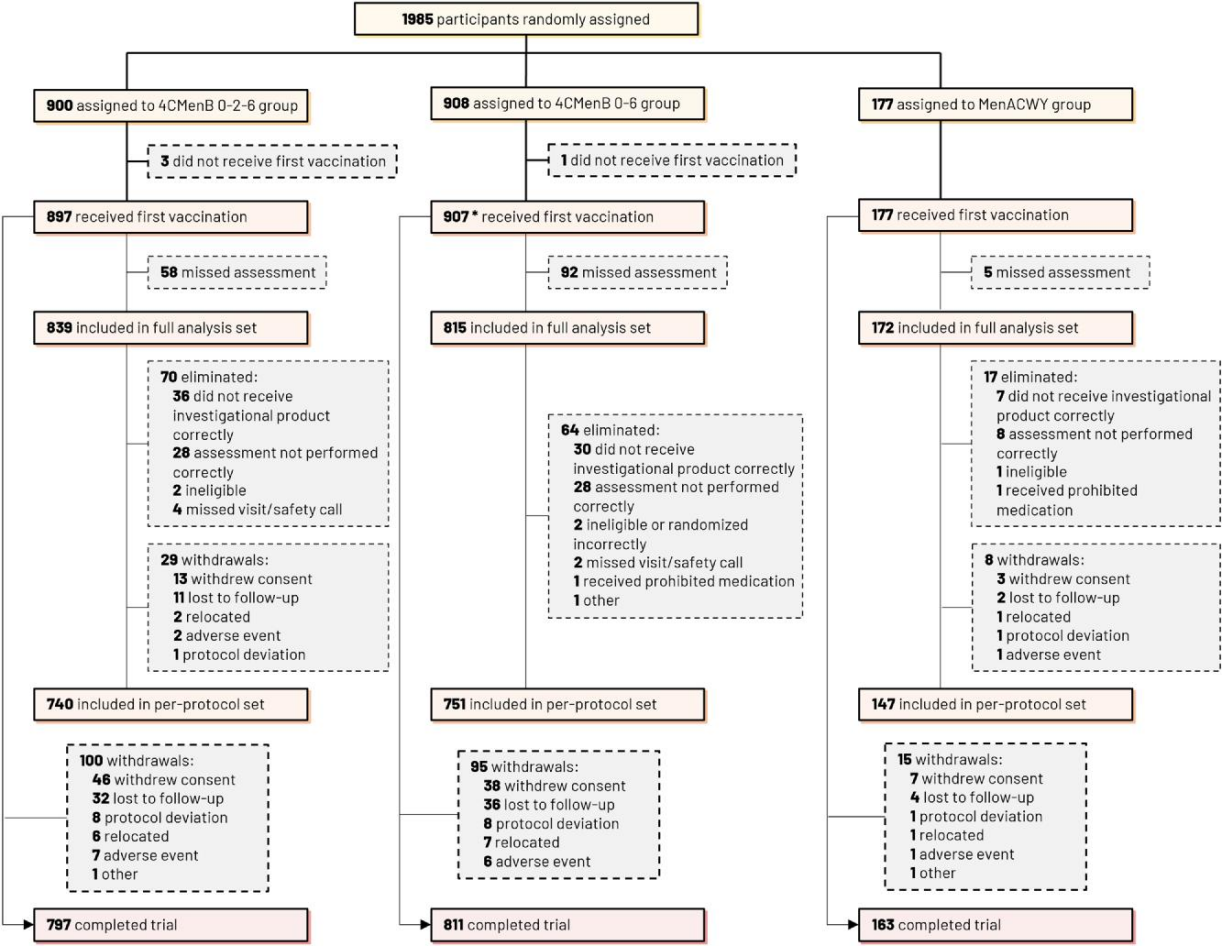
Trial profile of vaccinated study participants randomized to 4CMenB and MenACWY groups. *One participant was vaccinated at first study visit with MenACWY-CRM vaccine by human error and was subsequently vaccinated per the MenACWY group schedule. 4CMenB 0–2–6 group, 3 doses of 4-component meningococcal serogroup B (4CMenB) vaccine at study months 0, 2, 6; 4CMenB 0–6 group, 2 doses of 4CMenB at study months 0, 6; MenACWY group, meningococcal serogroups ACWY-CRM glycoconjugate vaccine.

Of 1985 adolescents and young adults randomized to receive 4CMenB or control MenACWY-CRM vaccination, 1981 (99.8%) received at least 1 study vaccine dose and of these, 1826 (92.0%) were included in the full analysis set and 1638 (82.5%) in the per-protocol set (Figure 2). The demographic and baseline characteristics of these participants were balanced between groups (Table 1). Overall, 1178 (59.3%) participants were aged 10 to 17 years and 807 (40.7%) were aged 18 to 25 years, 263 (13.2%) had received MenACWY vaccination, and 597 (30.1%) were from the US.

**Table 1.** Demographic Characteristics of Participants Randomized to the 4CMenB Groups or Control MenACWY Group

Characteristic	4CMenB 0–2–6 Group (N = 900)	4CMenB 0–6 Group (N = 908)	MenACWY Group (N = 177)
Age (y), mean (SD)	16.5 (4.7)	16.5 (4.7)	16.9 (4.6)
Age group, n (%)			
10–17 y	534 (59.3)	542 (59.7)	102 (57.6)
18–25 y	366 (40.7)	366 (40.3)	75 (42.4)
Sex, n (%)			
Male	434 (48.2)	461 (50.8)	77 (43.5)
Female	466 (51.8)	447 (49.2)	100 (56.5)
Race, n (%)			
White	799 (88.8)	793 (87.3)	161 (91.0)
Asian	43 (4.8)	60 (6.6)	9 (5.1)
Black or African American	33 (3.7)	29 (3.2)	6 (3.4)
Other	25 (2.8)	26 (2.9)	1 (0.6)
Previous MenACWY vaccination, n (%)	122 (13.6)	119 (13.1)	22 (12.4)

Abbreviations: 4CMenB 0–2–6 group, 3 doses of 4-component meningococcal serogroup B (4CMenB) vaccine at study months 0, 2, 6; 4CMenB 0–6 group, 2 doses of 4CMenB at study months 0, 6; MenACWY group, meningococcal serogroups ACWY-CRM glycoconjugate vaccine at month 0; previous MenACWY vaccination, vaccination with any MenACWY vaccine; N, number of participants in the group; n, number of participants in the category; SD, standard deviation; y, years.

### Breadth of Immune Response

**Figure 3.**
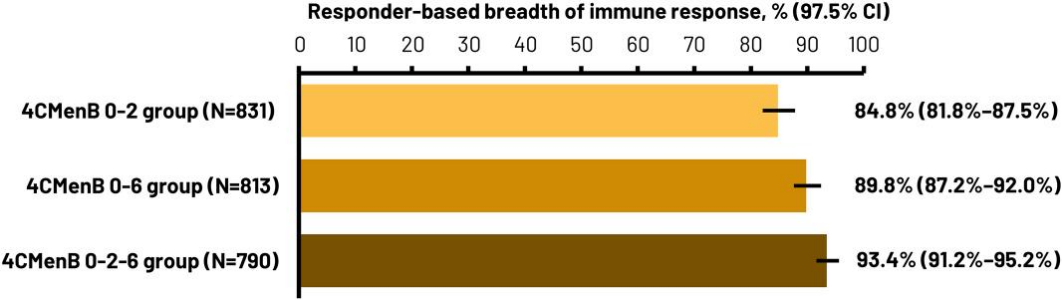
Responder-based analysis of breadth of immune response, measured by endogenous complement-human serum bactericidal antibody (enc-hSBA) assay against the 110 meningococcal serogroup B (MenB) strain panel, defined as percentage of participants whose sera kill ≥70% MenB strains at 1 month after last dose (full analysis set). Success was demonstrated if the lower limit of 2-sided 97.5% CI was above 65%. 4CMenB 0–2, 2 doses of 4-component meningococcal serogroup B (4CMenB) vaccine at study months 0, 2; 4CMenB 0–6, 2 doses of 4CMenB at study months 0, 6; 4CMenB 0–2–6, 3 doses of 4CMenB at study months 0, 2, 6; CI, confidence interval; N, number of participants.

Test-based (Table 2) and responder-based (Figure 3) breadth of immune response were demonstrated by enc-hSBA assay at 1 month after 2 and 3 4CMenB doses, with each lower limit of the 2-sided 97.5% CI above the predefined criterion of 65%. Test-based breadth of immune response was 78.7% (97.5% CI, 77.2–80.1), 81.8% (80.4–83.1), and 83.2% (81.9–84.4) for the 0–2, 0–6, and 0–2–6 schedules, respectively. Responder-based breadth of immune response was 84.8% (97.5% CI, 81.8–87.5), 89.8% (87.2–92.0), and 93.4% (91.2–95.2), respectively.

**Table 2.** Test-based Analysis of Breadth of Immune Response, Measured by enc-hSBA Assay Against the 110 MenB Strain Panel, Defined as Percentage of Samples Without Bactericidal Serum Activity Against the MenB Strain Panel After Last 4CMenB Dose Versus Percentage of Samples Without Bactericidal Serum Activity After a Single MenACWY-CRM Dose (per-protocol set)

Samples Without Bactericidal Serum Activity		Relative Risk (97.5% CI)	4CMenB Breadth Of Immune Response (97.5% CI)
4CMenB group	MenACWY group, N = 4374		
4CMenB 0–2 (N = 27 569): 16.8%	79.0%	.21 (.20, .23)	78.7% (77.2%, 80.1%)
4CMenB 0–6 (N = 26 142): 14.4%	79.0%	.18 (.17, .20)	81.8% (80.4%, 83.1%)
4CMenB 0–2–6 (N = 25 596): 13.3%	79.0%	.17 (.16, .18)	83.2% (81.9%, 84.4%)

For each sample, 35 strains were randomly selected for testing. Success was demonstrated if the lower limit of 2-sided 97.5% CI was >65%.

4CMenB 0–2, 2 doses of 4-component meningococcal serogroup B (4CMenB) vaccine at study months 0, 2; 4CMenB 0–6, 2 4CMenB doses at study months 0, 6; 4CMenB 0–2–6, 3 4CMenB doses at study months 0, 2, 6; MenACWY group, meningococcal serogroups ACWY-CRM glycoconjugate vaccine at month 0; CI, confidence interval; Enc-hSBA, endogenous complement-human serum bactericidal antibody; MenB, meningococcal serogroup B; N, number of samples.

### Immunogenicity

One month after 2-dose or 3-dose vaccination, the percentage of participants with a 4-fold rise in hSBA GMT from baseline against MenB indicator strains fHbp, *Neisseria* adhesin A, neisserial heparin-binding antigen, and Porin A was 74.6%, 96.3%, 58.5%, and 53.5%, respectively, with the 0–2 schedule, 82.4%, 95.3%, 69.5%, and 57.2%, respectively, with the 0–6 schedule, and 86.7%, 98.7%, 66.9%, and 56.5%, respectively, with the 0–2–6 schedule (Supplementary Table 1). The percentage of participants with hSBA titer ≥LLOQ against each indicator strain was also comparable among the 3 4CMenB schedules (Supplementary Table 2). For all indicator strains combined, the percentage with hSBA titer ≥ LLOQ was 75.5% (95% CI, 72.3–78.6), 80.7% (77.5–83.6), and 83.3% (80.3–86.1) for the 0–2, 0–6, and 0–2–6 schedule, respectively. A robust increase in hSBA GMT against each indicator strain was observed following all 4CMenB schedules (Supplementary Table 3).

### Safety

During the 7-day postvaccination period, solicited AEs were reported by 97.1% of participants in the 4CMenB 0–2–6 group, 95.8% in the 4CMenB 0–6 group, and 93.3% in the control MenACWY group. Overall percentages of participants reporting any, local, or systemic solicited AEs were similar among groups after each 4CMenB injection (Supplementary Table 4).

In all groups, pain at the injection site was the most frequently reported solicited local AE and fatigue and headache were the most frequently reported solicited systemic AEs (Supplementary Table 5). Most solicited AEs were mild to moderate in intensity, with severe AEs reported in <3% of participants in each group, apart from severe pain at the 4CMenB injection site, which was reported by 5.5% to 10.6% of participants after the first, second, and third dose in the 4CMenB 0–2–6 group, 5.6% and 8.3% after first and second dose in the 4CMenB 0–6 group, and 5.4% of the control group after 4CMenB administration at month 6 (Supplementary Table 5). Fever ≥40 °C was reported for 2 participants in the 4CMenB 0–2–6 group (after first and third doses), 2 participants in the 4CMenB 0–6 group (after first 4CMenB dose and MenACWY-CRM dose), and 1 participant in the MenACWY group (after MenACWY-CRM dose). The mean duration of solicited local or systemic AEs was <4 days (data not shown) and no increase in frequency of reporting was observed with subsequent doses of 4CMenB (Supplementary Table 5).

During the 30-day follow-up period after vaccination, similar percentages of participants in each group reported at least 1 unsolicited AE (29.6% in 4CMenB 0–2–6 group, 31.7% in 4CMenB 0–6 group, 29.8% in MenACWY group; Supplementary Table 6). The most commonly reported causally related unsolicited AE was injection site induration in the 4CMenB 0–2–6 group (8 participants) and MenACWY group (2 participants), and injection site pain (8 participants) in the 4CMenB 0–6 group.

Within 30 days of receiving a vaccine dose, SAEs were reported in 9 participants (1.0%) in the 4CMenB 0–2–6 group, 11 (1.2%) in the 4CMenB 0–6 group, and none in the control group (Supplementary Table 6). Five SAEs (nausea, vomiting, pyrexia, and headache in 1 participant and ulcerative colitis in 1 participant, both in the 4CMenB 0–6 group) were assessed as causally related to vaccination. The percentage of participants reporting a medically attended unsolicited AE was 14.2% in the 4CMenB 0–2–6 group, 17.2% in the 4CMenB 0–6 group, and 9.6% in the control group (Supplementary Table 6); each event had an incidence ≤2%. There were 2 deaths during the study, 1 from to poisoning (4CMenB 0–2–6 group) and 1 from intentional overdose (4CMenB 0–6 group), both considered not causally related to vaccination.

Eleven participants withdrew or were withdrawn because of an unsolicited AE (6 from 4CMenB 0–2–6 group, 4 from 4CMenB 0–6 group, 1 from MenACWY group). Of these, 7 experienced an SAE that led to study withdrawal, none of which were assessed as causally related to vaccination. Three nonserious AEs (arthritis in 1 participant in 4CMenB 0–2–6 group; pyrexia and injection site hematoma in 1 participant each in 4CMenB 0–6 group) leading to premature withdrawal were assessed as causally related to vaccination.

One participant in each 4CMenB group reported an adverse event of special interest, each classified as a new onset of a chronic disease: ulcerative colitis in the 4CMenB 0–6 group (SAE; onset 44 days after second dose) and arthritis in the 4CMenB 0–2–6 group (nonserious; onset 10 days after first 4CMenB dose). Both were coded by the site investigator as causally related to vaccination.

## DISCUSSION

This phase 3, multicenter, observer-blind study investigated breadth of immune response, immunogenicity, and safety for 4CMenB in adolescents and young adults when administered in a 2-dose (0–2 or 0–6 months) or 3-dose (0–2–6 months) schedule. Evaluation of breadth of immune response was by enc-hSBA assay of a panel of 110 genetically diverse MenB strains representative of global circulating strains, in terms of clonal complex distribution and MenB vaccine antigen genotypes [34]. Point estimates for 4CMenB breadth of immune response, whether administered as 2 doses 2 or 6 months apart or as 3 doses over 6 months, were 79%–83% (test-based, reflecting the risk reduction in getting infected by a MenB strain) and 85%–93% (responder-based, reflecting the proportion of individuals who mount a killing response against the majority of tested MenB strains). The predefined success criterion was met for both breadth of immune response endpoints for all 4CMenB schedules. Because no clinically relevant increase in breadth of immune response or immunogenicity was observed with the 3-dose schedule or with a dose interval of 6 months, these results support use of the 2-dose 0–2 months schedule, which is particularly relevant for outbreak control and prevention.

The enc-hSBA assay was developed to assess the ability of a multicomponent vaccine to induce a bactericidal immune response against a broad panel of genetically diverse MenB strains in clinical trial settings. Through the use of endogenous complement, it accounts for natural heterogeneity in complement killing activity as well as the synergistic effects of antibodies elicited by multiple MenB vaccine antigens against the variety of diverse circulating strains [29, 34]. This enables breadth of immune response to be measured in a clinical trial under conditions that approximate real-world settings [8] and may therefore predict real-world vaccine performance. This is supported by the observation that point values following 2-dose (0–2 months) 4CMenB in this study of adolescents and young adults (78.7% and 84.8%) are in line with real-world evidence of the impact of 4CMenB vaccination, specifically a 78.5% reduction in MenB disease incidence and 83.5% vaccine effectiveness against MenB disease in adolescents in the 3 years after a 2-dose 4CMenB vaccination program was implemented in South Australia [22].

Results with the traditional hSBA assay show 4CMenB induced robust immune responses against each MenB indicator strain, with no clinically meaningful differences in immunogenicity among schedules. This is consistent with a previous study in adolescents that found a third 4CMenB dose provided no additional immunological benefit [40]. 4CMenB was well tolerated in all schedules, with overall no increase in the frequency of solicited AEs with subsequent doses, and no new safety concerns identified. Based on reported information, definitive causality attribution was not possible for the 2 adverse events of special interest classified as a new onset of a chronic condition and coded as causally related to vaccination by the site investigator. The safety profile of 4CMenB in this study was in line with observations from previous clinical studies and real-world safety studies of the vaccine administered to adolescents and young adults [41].

Study limitations include the 10- to 25-year age group assessed, which might reduce the generalizability of the results to other age groups. However, previous studies show the immune response following 4CMenB vaccination in older or younger individuals is broadly comparable to that in adolescents and young adults [42, 43] and confirm the acceptable safety profile of 4CMenB across various age groups [41].

## CONCLUSIONS

We assessed the 4CMenB breadth of immune response against an epidemiologically relevant panel of 110 MenB strains using the enc-hSBA assay. The 4CMenB primary objectives of this study were met, with results confirming the performance of 0–2, 0–6, and 0–2–6 dose schedules against diverse disease-causing MenB strains, in line with real-world evidence of 4CMenB impact and effectiveness, and safety results consistent with the established safety profile of 4CMenB. All 4CMenB schedules induced robust immune responses against each MenB indicator strain. Because there were no clinically meaningful differences in breadth of immune response or immunogenicity among schedules, the results show a third 4CMenB dose or extending the dose interval to 6 months provides no additional immunological benefit over the 0–2 months schedule, which is especially important for outbreak control. These results also support use of the enc-hSBA assay against a broad MenB strain panel for assessment of breadth of immune response for multicomponent MenB vaccines in clinical trials in conditions that are close to real-world settings.

## Supplementary Data

Supplementary materials are available at *Open Forum Infectious Diseases* online. Consisting of data provided by the authors to benefit the reader, the posted materials are not copyedited and are the sole responsibility of the authors, so questions or comments should be addressed to the corresponding author.

## Supplementary Material

ofae638_Supplementary_Data

## Notes

***Acknowledgments***. The authors thank the participants, site personnel and investigators who took part in the study. The authors also thank GSK project team members for their contribution to the study and/or development of this manuscript. The authors also thank Business & Decision Life Sciences Medical Communication Service Center for editorial assistance, manuscript coordination and Joanne Knowles (independent medical writer) for medical writing support, on behalf of GSK.

***Author Contributions***. Conceptualization: C.B., M.L., and D.T. Data curation: A.W. Formal analysis: A.W. Investigation: J.B., M.B., B.S.C., E.C.D., D.D., S. Kokko, S. Koski, O.L., J.M.L., M.R., P.C.R., P.S., B.T., F.T., M.T., B. Ukkonen, and B. Ulukol. Methodology: C.B., M.L., A.W., and D.T. Project administration: all authors. Resources: all authors. Software: A.W. Supervision: C.B., M.L., and D.T. Validation: T.N., C.B., P.S., M.L., M.T., A.W., and D.T. Visualization: T.N., C.B., P.S., M.L., M.T., A.W., and D.T. Writing of the original draft: T.N., C.B., M.L., M.T., A.W., and D.T. Review and editing of draft: all authors.

***Data availability***. Please refer to GSK weblink to access GSK's data sharing policies and, as applicable, seek anonymized subject level data via the link https://www.gsk-studyregister.com/en/.

***Financial support*.** GSK funded this research and was involved in all stages of research conduct, including analysis of the data. GSK also took in charge all costs associated with the development and publication of this manuscript.

***Trademarks***. Bexsero and Menveo are trademarks owned by or licensed to GSK. Trumenba is a trademark of Pfizer.
