# Supplementary material for: 4CMenB Breadth of Immune Response, Immunogenicity, and Safety: Results From a Phase 3 Randomized, Controlled, Observer Blind Study in Adolescents and Young Adults
Source: Open Forum Infect Dis. 2024 Oct 30;11(11):ofae638. doi: 10.1093/ofid/ofae638 (PMC11584413; doi:10.1093/ofid/ofae638)
Supplement: ofae638_Supplementary_Data [file ofae638_supplementary_data.docx]

**SUPPLEMENTARY TEXT**

**QUINTET Study Group**

Ronald Ackerman, Renata Adamovska, Eugene Athan, Kwabena Ayesu, Jiří Beran, David Bernard, Chiranjiwi Bhusal, Mark Bloch, William Byars, Robert Carter, Benhur Cetin, Maia Chakerian, Marije Dalebout, Ferdinandus de Looze, Ener Cagri Dinleyici, Marc Dionne, Daniel Dražan, Peter Dzongowski, Rand Farjo, Daniel Finn, George Freeman, Ryan Gottfredson, Paul Grubb, Anil Gupta, Tolga Ince, Robert Jeanfreau, Jake Jones, James Kellner, Kaia Kiiroja, Satu Kokko, Susanna Koski, Joanne Langley, Outi Laajalahti, Maria Lattanzi, Isabelle Lechevin, Hemalini Mehta, Sandra Meisalu, Danielle Morelle, Terry Nolan, Alexander Osowa, Pauliina Paavola, Minesh Patel, Miroslav Pavlasek, Enrique Pelayo, Mika Rämet, Stefanie Raulier, Peter Richmond, Walter Rok, Rambod Rouhbakhsh, Manish Sadarangani, Yamirka Sanchez, Martin Schear, John Scott, Ilkka Seppä, Peter Silas, William Simon, Martina Spaziererova, Jonathan Staben, Joseph Surber, Bruce Tapiero, Florence Tiong, Mary Tipton, Daniela Toneatto, Mauro Trapani, Benita Ukkonen, Betul Ulukol, Marie-Louise Vachon, Noah Vale, Dominique Wauters, Arnold Willemsen, Josef Zemanek

| **First Name** | **Last Name** | **Institution** | **City** | **Country** | **Email** |
| --- | --- | --- | --- | --- | --- |
| Mark | Bloch | Holdsworth House Medical Practice, and University of New South Wales | Sydney | Australia | mark.bloch@holdsworthhouse.com.au |
| Peter | Richmond | Telethon Kids Institute | Nedlands | Australia | peter.richmond@uwa.edu.au |
| Eugene | Athan | Barwon Health - University Hospital Geelong | Geelong | Australia | eugene@barwonhealth.org.au |
| Terry | Nolan | Peter Doherty Institute at the University of Melbourne, and Murdoch Children’s Research Institute | Melbourne | Australia | t.nolan@unimelb.edu.au |
| Marije | Dalebout | Griffith University | Gold Coast | Australia | marije.dalebout@gmail.com |
| Florence | Tiong | AusTrials (Wellers Hill) | Tarragindi | Australia | florence.tiong@austrials.com.au |
| Ferdinandus | de Looze | AusTrials (Taringa) | Taringa | Australia | fred.delooze@austrials.com.au |
| Ener | Dinleyici | Eskisehir Osmangazi University Faculty of Medicine | Eskisehir | Türkiye | enercagri@gmail.com |
| Tolga | Ince | Dokuz Eylul University Medical Faculty | Izmir | Türkiye | drtince@gmail.com |
| Benhur | Cetin | Erciyes University Faculty of Medicine | Kayseri | Türkiye | benhurcetin@yahoo.com |
| Betul | Ulukol | Ankara University School Of Medicine | Ankara | Türkiye | betul.ulukol@gmail.com |
| Jiří | Beran | Vaccination and Travel Medicine Centre | Hradec Králové | Czechia | jiri.beran@vakcinace.cz |
| Daniel | Dražan | General Practice for Children and Adolescents | Jindřichův Hradec | Czechia | daniel@danieldrazan.cz |
| Renata | Adamovska | Prakticky lekar pro deti a dorost | Praha 6 | Czechia | drruzkova@email.cz |
| Martina | Spaziererova | Ordinace praktickeho lekare pro deti a dorost | Kladno | Czechia | martina.spaziererova@eucklinika.cz |
| Josef | Zemanek | Ordinace praktickeho lekare pro deti a dorost | Tynec nad Sazavou | Czechia | josef.zemanek@detskylekar.com |
| Miroslav | Pavlasek | Admed s.r.o | Ceske Budejovice | Czechia | dr.pavlasek@centrum.cz |
| Sandra | Meisalu | Innomedica OU | Tallinn | Estonia | [sandra.meisalu@innomedica.ee](mailto:sandra.meisalu@innomedica.ee) |
| Kaia | Kiiroja | Al Mare Family Doctor Centre | Tallinn | Estonia | kaia@almarearstid.ee |
| Mika | Rämet | Tampereen yliopisto Tampereen rokotetutkimusklinikka | Tampere | Finland | mika.ramet@fvr.fi |
| Ilkka | Seppa | Tampereen yliopisto Turun rokotetutkimusklinikka | Turku | Finland | ilkka.seppa@fvr.fi |
| Susanna | Koski | Tampereen yliopisto Etelä-Helsingin rokotetutkimusklinikka, | Helsinki | Finland | susanna.koski@fvr.fi |
| Benita | Ukkonen | Tampereen yliopisto Espoon rokotetutkimusklinikka | Espoo | Finland | benita.ukkonen@fvr.fi |
| Satu | Kokko | Tampereen yliopisto Oulun rokotetutkimusklinikka | Oulu | Finland | satu.kokko@fvr.fi |
| Outi | Laajalahti | Tampereen yliopisto Seinäjoen rokotetutkimusklinikka | Seinäjoki | Finland | outi.laajalahti@fvr.fi |
| Pauliina | Paavola | Tampereen yliopisto Kokkolan rokotetutkimusklinikka | Kokkola | Finland | pauliina.paavola@fvr.fi |
| Marc | Dionne | Equipe de recherche en vaccination du Centre de recherche du CHU de Quebec | Quebec City | Canada | marc.dionne@crchudequebec.ulaval.ca |
| Anil | Gupta | Dr. Anil Gupta and Associates | Toronto | Canada | anil@albionfinchmedical.com |
| Joanne | Langley | Canadian Center for Vaccinology, Dalhousie University | Halifax | Canada | joanne.langley@dal.ca |
| James | Kellner | Alberta Children's Hospital | Calgary | Canada | jim.kellner@albertahealthservices.ca |
| Bruce | Tapiero | CHU Sainte-Justine, and University of Montreal | Montreal | Canada | bruce.tapiero.hsj@ssss.gouv.qc.ca |
| Noah | Vale | Manna Research | Toronto | Canada | valeresearch@lmcmanna.com |
| Marie-Louise | Vachon | CHU de Québec-Université Laval | Quebec | Canada | marie-louise.vachon.med@ssss.gouv.qc.ca |
| Manish | Sadarangani | B.C. Research Institute for Children's and Women's Health | Vancouver | Canada | msadarangani@bcchr.ubc.ca |
| Peter | Dzongowski | Milestone Research | London | Canada | drpeter@milestoneresearch.ca |
| Mary | Tipton | Copperview Medical Center | South Jordan | USA | mary.tipton@amrllc.com |
| Walter | Rok | Pediatric Associates of Fall River | Fall River | USA | wjrok@cox.net |
| Martin | Schear | Dayton Clinical Research | Dayton | USA | mschear@aol.com |
| William | Simon | Alliance for Multispecialty Research LLC | Wichita | USA | william.simon@amrllc.com |
| Jonathan | Staben | Multicare Rockwood Cheney Clinic | Cheney | USA | jstaben@multicare.org |
| Paul | Grubb | MultiCare Institute for Research & Innovation | Spokane | USA | pgrubb@multicare.org |
| Robert | Jeanfreau | Medpharmics | Metairie | USA | robertjeanfreau@medpharmics.com |
| Enrique | Pelayo | Advanced Medical Research Institute | Miami | USA | epelayomd@amriresearch.com |
| Minesh | Patel | East Coast Institute for Research | Lake City | USA | drmpatel2@gmail.com |
| Rambod | Rouhbakhsh | MediSync Clinical Research Hattiesburg Clinic | Petal | USA | rambod.rouhbakhsh@hattiesburgclinic.com |
| Peter | Silas | Wee Care Pediatrics | Syracuse | USA | peter.silas@amrllc.com |
| Daniel | Finn | Kentucky Pediatric/Adult Research | Bardstown | USA | danieljfinn@yahoo.com |
| Joseph | Surber | IACT Health | Columbus | USA | joe.surber@centricityresearch.com |
| George | Freeman | Health Research of Hampton Roads, Inc. | Newport News | USA | ghfreeman@hrhr-inc.com |
| Maia | Chakerian | Samaritan Center for Medical Research Medical Group | Los Gatos | USA | mchakerian@samaritan-research.com |
| Alexander | Osowa | Gwinnett Research Institute | Buford | USA | alex.osowa@gwinnettresearchinstitute.com |
| Kwabena | Ayesu | Omega Research Orlando LLC | Orlando | USA | kayesu@omegarcllc.com |
| Jake | Jones | Cottonwood Pediatrics | Murray | USA | jones@rx-research.com |
| Ryan | Gottfredson | Utah Valley Pediatrics Timpanogos Office | Orem | USA | gottfredson@rx-research.com |
| John | Scott | National Clinical Research | Richmond | USA | kscott@ncrinc.net |
| William | Byars | Tribe Clinical Research LLC | Greenville | USA | wbyars@mvcresearch.com |
| Hemalini | Mehta | Clinical Research Institute | Minneapolis | USA | hm@allergy-asthma-docs.com |
| Robert | Carter | Coastal Heritage Clinical Research | Hinesville | USA | drcarterchcr@gmail.com |
| Rand | Farjo | Ascension St John Hospital | Grosse Pointe Woods | USA | [rand.farjo@ascension.org](mailto:rand.farjo@ascension.org) |
| Ronald | Ackerman | Comprehensive Clinical Trials, LLC | West Palm Beach | USA | gobs@earthlink.net |
| David | Bernard | CHEAR Center LLC | Bronx | USA | david@chearcenter.com |
| Yamirka | Sanchez | Oceane7 Medical & Research Center Inc | Miami | USA | y.sanchez@oceane7.us |
| Maria | Lattanzi | GSK | Siena | Italy | maria.x.lattanzi@gsk.com |
| Mauro | Trapani | GSK | Siena | Italy | mauro.x.trapani@gsk.com |
| Chiranjiwi | Bhusal | GSK | Amsterdam | The Netherlands | chiranjiwi.x.bhusal@gsk.com |
| Daniela | Toneatto | GSK | Siena | Italy | daniela.x.toneatto@gsk.com |
| Arnold | Willemsen | GSK | Amsterdam | The Netherlands | arnold.x.willemsen@gsk.com |
| Isabelle | Lechevin | GSK | Wavre | Belgium | isabelle.lechevin@gsk.com |
| Stefanie | Raulier | GSK | Wavre | Belgium | stefanie.x.raulier@gsk.com |
| Dominique | Wauters | GSK | Wavre | Belgium | dominique.wauters@gsk.com |
| Danielle | Morelle | GSK | Wavre | Belgium | danielle.morelle@gsk.com |

**Definitions of four-fold rise in hSBA titer, LOD, and LLOQ**

A four-fold rise in human serum bactericidal antibody (hSBA) titer was defined as a post-vaccination hSBA titer at least four times the limit of detection (LOD) for participants with a pre-vaccination hSBA titer <LOD, a post-vaccination hSBA titer at least four times the lower limit of quantitation (LLOQ) for participants with a pre-vaccination hSBA titer ≥LOD and < LLOQ, and a post-vaccination hSBA titer at least four times the pre-vaccination hSBA titer for participants with a pre-vaccination hSBA titer ≥LLOQ. The LOD was 3 for factor H binding protein (fHbp), 6 for *Neisseria* adhesin A (NadA), and 4 for neisserial heparin-binding antigen (NHBA) and Porin A (PorA). The LLOQ was 5 for fHbp, 15 for NadA, 4 for NHBA, and 6 for PorA.

**Supplementary Table 1.** Percentages of participants with four-fold rise from baseline in human serum bactericidal antibody (hSBA) geometric mean titer (GMT) against each meningococcal serogroup B (MenB) indicator strain after 4CMenB 0-2-6 months schedule (month 7), 0-2 months schedule (0-2-6 group, month 3), and 0-6 months schedule (month 7) (full analysis set)

| **MenB indicator strain**  **Timepoint** | **Percentage of participants with 4-fold increase in hSBA GMT (95% CI)** | |
| --- | --- | --- |
|  | **4CMenB 0-2-6 group** | **4CMenB 0-6 group** |
| fHbp |  |  |
| Month 3 | 74.6 (71.3–77.7) |  |
| Month 7 | 86.7 (84.0–89.2) | 82.4 (79.4–85.2) |
| NadA |  |  |
| Month 3 | 96.3 (94.7–97.6) |  |
| Month 7 | 98.7 (97.5–99.4) | 95.3 (93.4–96.7) |
| NHBA |  |  |
| Month 3 | 58.5 (54.8–62.0) |  |
| Month 7 | 66.9 (63.2–70.4) | 69.5 (65.9–72.8) |
| PorA |  |  |
| Month 3 | 53.5 (49.7–57.1) |  |
| Month 7 | 56.5 (52.6–60.4) | 57.2 (53.4–61.0) |

4CMenB 0-2-6 group, three doses of 4-component meningococcal serogroup B (4CMenB) vaccine at study months 0, 2, 6; 4CMenB 0-6 group, two doses of 4CMenB at study months 0, 6; CI, confidence interval; fHbp, factor H binding protein; NadA, *Neisseria* adhesin A; NHBA, neisserial heparin-binding antigen; PorA, Porin A.

Total number of participants: 4CMenB 0-2-6 group, 724–739 at month 3, 637–685 at month 7; 4CMenB 0-6 group, 664–704 at month 7.

**Supplementary Table 2.** Percentages of participants at each timepoint with human serum bactericidal antibody (hSBA) titer ≥ lower limit of quantitation (LLOQ) at baseline and after 4CMenB 0-2-6 months schedule (month 7), 0-2 months schedule (0-2-6 group, month 3), and 0-6 months schedule (month 7) (full analysis set)

| **MenB indicator strain**  **Timepoint** | **Percentage of participants with hSBA titer ≥ LLOQ  (95% CI)** | |
| --- | --- | --- |
|  | **4CMenB 0-2-6 group** | **4CMenB 0-6 group** |
| fHbp |  |  |
| Baseline | 4.9 (3.5–6.7) | 3.4 (2.2–5.0) |
| Month 3 | 92.9 (90.9–94.7) |  |
| Month 7 | 97.4 (95.9–98.4) | 94.6 (92.7–96.2) |
| NadA |  |  |
| Baseline | 6.2 (4.6–8.2) | 4.4 (3.0–6.1) |
| Month 3 | 99.5 (98.6–99.9) |  |
| Month 7 | 100 (99.5–100) | 98.0 (96.7–98.9) |
| NHBA |  |  |
| Baseline | 23.2 (20.3–26.4) | 20.9 (18.0–24.1) |
| Month 3 | 96.1 (94.5–97.4) |  |
| Month 7 | 97.0 (95.4–98.1) | 97.5 (96.0–98.5) |
| PorA |  |  |
| Baseline | 2.3 (1.3–3.7) | 1.4 (0.7–2.6) |
| Month 3 | 80.0 (76.9–82.8) |  |
| Month 7 | 85.8 (82.9–88.4) | 82.6 (79.5–85.4) |
| All MenB indicator strains |  |  |
| Baseline | 1.1 (0.5–2.2) | 0.6 (0.2–1.4) |
| Month 3 | 75.5 (72.3–78.6) |  |
| Month 7 | 83.3 (80.3–86.1) | 80.7 (77.5–83.6) |

4CMenB 0-2-6 group, three doses of 4-component meningococcal serogroup B (4CMenB) vaccine at study months 0, 2, 6; 4CMenB 0-6 group, two doses of 4CMenB at study months 0, 6; CI, confidence interval; fHbp, factor H binding protein; MenB, meningococcal serogroup B; NadA, *Neisseria* adhesin A; NHBA, neisserial heparin-binding antigen; PorA, Porin A.

Total number of participants: 4CMenB 0-2-6 group, 727–749 at baseline, 744–753 at month 3, 654–695 at month 7; 4CMenB 0-6 group, 708–731 at baseline, 683–711 at month 7.

**Supplementary Table 3.** Human serum bactericidal antibody (hSBA) geometric mean titers (GMTs) against each meningococcal serogroup B (MenB) indicator strain at baseline and after 4CMenB 0-2-6 months schedule (month 7), 0-2 months schedule (0-2-6 group, month 3), and 0-6 months schedule (month 7) (full analysis set)

| **MenB indicator strain**  **Timepoint** | **hSBA GMT (95% CI)** | |
| --- | --- | --- |
|  | **4CMenB 0-2-6 group** | **4CMenB 0-6 group** |
| fHbp |  |  |
| Baseline | 2.8 (2.7–2.8) | 2.7 (2.6–2.8) |
| Month 3 | 20.9 (18.9–23.1) |  |
| Month 7 | 30.8 (28.3–33.5) | 28.1 (25.9–30.6) |
| NadA |  |  |
| Baseline | 8.4 (8.1–8.6) | 8.3 (8.0–8.6) |
| Month 3 | 178.5 (161.7–197.2) |  |
| Month 7 | 267.2 (243.7–293.0) | 215.1 (196.2–235.9) |
| NHBA |  |  |
| Baseline | 3.4 (3.1–3.7) | 3.2 (3.0–3.5) |
| Month 3 | 27.2 (24.1–30.6) |  |
| Month 7 | 30.6 (27.7–33.7) | 33.2 (30.2–36.6) |
| PorA |  |  |
| Baseline | 3.2 (3.1–3.2) | 3.1 (3.0–3.2) |
| Month 3 | 17.1 (15.2–19.3) |  |
| Month 7 | 18.1 (16.3–20.1) | 17.7 (15.9–19.6) |

4CMenB 0-2-6 group, three doses of 4-component meningococcal serogroup B (4CMenB) vaccine at study months 0, 2, 6; 4CMenB 0-6 group, two doses of 4CMenB at study months 0, 6; CI, confidence interval; fHbp, factor H binding protein; NadA, *Neisseria* adhesin A; NHBA, neisserial heparin-binding antigen; PorA, Porin A.

Total number of samples: 4CMenB 0-2-6 group, 738–749 at baseline, 745–753 at month 3, 657–695 at month 7; 4CMenB 0-6 group, 716–731 at baseline, 684–711 at month 7.

**Supplementary Table 4.** Summary of numbers and percentages of participants reporting solicited local and systemic adverse events (AEs) within 7 days of each injection (solicited safety set)

|  | **4CMenB 0-2-6 group, n (%)** | **4CMenB 0-6 group, n (%)** | **MenACWY group, n (%)** |
| --- | --- | --- | --- |
| Injection 1 | N=885 | N=894 | N=178 |
| Any AE | 828 (93.6) | 837 (93.6) | 126 (70.8) |
| Local AE | 812 (91.8) | 824 (92.2) | 73 (41.0) |
| Systemic AE | 563 (63.6) | 550 (61.5) | 106 (59.6) |
| Injection 2 | N=823 | N=813 | N=161 |
| Any AE | 741 (90.0) | 436 (53.6) | 72 (44.7) |
| Local AE | 720 (87.5) | 235 (28.9) | 32 (19.9) |
| Systemic AE | 473 (57.5) | 341 (41.9) | 58 (36.0) |
| Injection 3 | N=765 | N=759 | N=148 |
| Any AE | 700 (91.5) | 688 (90.6) | 130 (87.8) |
| Local AE | 681 (89.0) | 678 (89.3) | 126 (85.1) |
| Systemic AE | 479 (62.6) | 441 (58.1) | 75 (50.7) |
| Any injection | N=890 | N=900 | N=178 |
| Any AE | 864 (97.1) | 862 (95.8) | 166 (93.3) |
| Local AE | 853 (95.8) | 848 (94.2) | 148 (83.1) |
| Systemic AE | 722 (81.1) | 703 (78.1) | 133 (74.7) |

4CMenB 0-2-6 group, 4-component meningococcal serogroup B (4CMenB) vaccine at each injection; 4CMenB 0-6 group, administered 4CMenB at injections 1 and 3, meningococcal serogroups ACWY-CRM glycoconjugate vaccine (MenACWY-CRM) at injection 2; MenACWY group, administered MenACWY-CRM at injection 1, placebo at injection 2, 4CMenB at injection 3; N, number of participants in group who provided solicited AE data; n, number of participants in solicited AE category.

**Supplementary Table 5.** Percentages of participants reporting solicited local and systemic adverse events within 7 days of vaccination (solicited safety set)

|  |  | **Percentage of participants (95% CI)** | | |
| --- | --- | --- | --- | --- |
| **Adverse event** | **Intensity** | **4CMenB 0-2-6 group** | **4CMenB 0-6 group** | **MenACWY group** |
| Pain |  |  |  |  |
| Injection 1 | Any | 91.2 (89.1–93.0) | 91.6 (89.6–93.3) | 37.6 (30.5–45.2) |
|  | Severe | 5.5 (4.1–7.3) | 5.6 (4.2–7.3) | 0 |
| Injection 2 | Any | 86.8 (84.2–89.0) | 27.6 (24.5–30.8) | 18.6 (12.9–25.5) |
|  | Severe | 6.9 (5.3–8.9) | 0.6 (0.2–1.4) | 0 |
| Injection 3 | Any | 88.5 (86.0–90.7) | 89.1 (86.6–91.2) | 85.1 (78.4–90.4) |
|  | Severe | 10.6 (8.5–13.0) | 8.3 (6.4–10.5) | 5.4 (2.4–10.4) |
| Erythema |  |  |  |  |
| Injection 1 | Any | 10.2 (8.3–12.4) | 9.6 (7.8–11.7) | 6.2 (3.1–10.8) |
|  | Severe | 0.9 (0.4–1.8) | 0.8 (0.3–1.6) | 1.1 (0.1–4.0) |
| Injection 2 | Any | 10.8 (8.8–13.1) | 3.2 (2.1–4.7) | 0.6 (0.0–3.4) |
|  | Severe | 1.3 (0.7–2.4) | 0.5 (0.1–1.3) | 0 |
| Injection 3 | Any | 15.4 (12.9–18.2) | 11.5 (9.3–13.9) | 7.4 (3.8–12.9) |
|  | Severe | 2.2 (1.3–3.5) | 1.3 (0.6–2.4) | 0.7 (0.0–3.7) |
| Swelling |  |  |  |  |
| Injection 1 | Any | 9.8 (7.9–12.0) | 10.0 (8.1–12.1) | 6.2 (3.1–10.8) |
|  | Severe | 0.8 (0.3–1.6) | 1.0 (0.5–1.9) | 1.1 (0.1–4.0) |
| Injection 2 | Any | 12.0 (9.9–14.4) | 2.7 (1.7–4.1) | 0.6 (0.0–3.4) |
|  | Severe | 1.0 (0.4–1.9) | 0.4 (0.1–1.1) | 0 |
| Injection 3 | Any | 14.0 (11.6–16.6) | 11.2 (9.0–13.7) | 8.8 (4.8–14.6) |
|  | Severe | 1.2 (0.5–2.2) | 1.8 (1.0–3.1) | 0.7 (0.0–3.7) |
| Induration |  |  |  |  |
| Injection 1 | Any | 6.8 (5.2–8.6) | 7.2 (5.6–9.1) | 3.9 (1.6–7.9) |
|  | Severe | 1.0 (0.5–1.9) | 1.6 (0.9–2.6) | 0 |
| Injection 2 | Any | 8.1 (6.4–10.2) | 2.3 (1.4–3.6) | 0 |
|  | Severe | 1.0 (0.4–1.9) | 0.6 (0.2–1.4) | 0 |
| Injection 3 | Any | 6.8 (5.1–8.8) | 7.5 (5.7–9.6) | 8.1 (4.3–13.7) |
|  | Severe | 1.3 (0.6–2.4) | 1.1 (0.5–2.1) | 1.4 (0.2–4.8) |
| Fatigue |  |  |  |  |
| Injection 1 | Any | 47.8 (44.5–51.1) | 46.3 (43.0–49.6) | 43.8 (36.4–51.4) |
|  | Severe | 1.6 (0.9–2.6) | 1.2 (0.6–2.2) | 2.2 (0.6–5.7) |
| Injection 2 | Any | 45.2 (41.8–48.7) | 28.0 (25.0–31.3) | 22.4 (16.2–29.6) |
|  | Severe | 1.8 (1.0–3.0) | 0.7 (0.3–1.6) | 1.9 (0.4–5.3) |
| Injection 3 | Any | 48.9 (45.3–52.5) | 44.9 (41.3–48.5) | 37.8 (30.0–46.2) |
|  | Severe | 2.9 (1.8–4.3) | 2.6 (1.6–4.0) | 2.7 (0.7–6.8) |
| Nausea |  |  |  |  |
| Injection 1 | Any | 12.7 (10.5–15.0) | 12.4 (10.3–14.8) | 15.2 (10.2–21.3) |
|  | Severe | 0.5 (0.1–1.2) | 0.7 (0.2–1.5) | 1.1 (0.1–4.0) |
| Injection 2 | Any | 12.6 (10.4–15.1) | 6.9 (5.2–8.9) | 11.2 (6.8–17.1) |
|  | Severe | 0.6 (0.2–1.4) | 0.1 (0.0–0.7) | 1.2 (0.2–4.4) |
| Injection 3 | Any | 12.3 (10.0–14.8) | 11.1 (8.9–13.5) | 9.5 (5.3–15.4) |
|  | Severe | 0.3 (0.0–0.9) | 0.4 (0.1–1.2) | 0.7 (0.0–3.7) |
| Myalgia |  |  |  |  |
| Injection 1 | Any | 10.4 (8.5–12.6) | 11.9 (9.8–14.2) | 7.3 (3.9–12.2) |
|  | Severe | 0.2 (0.0–0.8) | 0.7 (0.2–1.5) | 0 |
| Injection 2 | Any | 13.4 (11.1–15.9) | 5.7 (4.2–7.5) | 1.9 (0.4–5.3) |
|  | Severe | 0.6 (0.2–1.4) | 0.2 (0.0–0.9) | 0 |
| Injection 3 | Any | 13.9 (11.5–16.5) | 14.4 (11.9–17.1) | 11.5 (6.8–17.8) |
|  | Severe | 1.2 (0.5–2.2) | 0.4 (0.1–1.2) | 0 |
| Arthralgia |  |  |  |  |
| Injection 1 | Any | 6.3 (4.8–8.1) | 7.8 (6.2–9.8) | 9.6 (5.7–14.9) |
|  | Severe | 0.2 (0.0–0.8) | 0.3 (0.1–1.0) | 0 |
| Injection 2 | Any | 8.7 (6.9–10.9) | 4.1 (2.8–5.7) | 3.7 (1.4–7.9) |
|  | Severe | 0.6 (0.2–1.4) | 0 | 0 |
| Injection 3 | Any | 9.3 (7.3–11.6) | 7.0 (5.3–9.0) | 4.7 (1.9–9.5) |
|  | Severe | 0.4 (0.1–1.1) | 0 | 0 |
| Headache |  |  |  |  |
| Injection 1 | Any | 40.5 (37.2–43.8) | 36.9 (33.7–40.2) | 38.8 (31.6–46.3) |
|  | Severe | 1.2 (0.6–2.2) | 1.1 (0.5–2.0) | 2.2 (0.6–5.7) |
| Injection 2 | Any | 36.6 (33.3–40.0) | 27.4 (24.4–30.6) | 19.3 (13.5–26.2) |
|  | Severe | 2.3 (1.4–3.6) | 0.7 (0.3–1.6) | 0.6 (0.0–3.4) |
| Injection 3 | Any | 39.5 (36.0–43.0) | 37.4 (34.0–41.0) | 26.4 (19.5–34.2) |
|  | Severe | 2.4 (1.4–3.7) | 1.1 (0.5–2.1) | 1.4 (0.2–4.8) |
| Fever |  |  |  |  |
| Injection 1 | Any | 2.1 (1.3–3.3) | 1.9 (1.1–3.0) | 1.7 (0.3–4.8) |
|  | Severe | 0.1 (0.0–0.6) | 0.1 (0.0–0.6) | 0.6 (0.0–3.1) |
| Injection 2 | Any | 2.7 (1.7–4.0) | 1.5 (0.8–2.6) | 0.6 (0.0–3.4) |
|  | Severe | 0 | 0.1 (0.0–0.7) | 0 |
| Injection 3 | Any | 2.7 (1.7–4.2) | 3.0 (1.9–4.5) | 1.4 (0.2–4.8) |
|  | Severe | 0.1 (0.0–0.7) | 0 | 0 |

CI, confidence interval.

4CMenB 0-2-6 group, administered 4-component meningococcal serogroup B (4CMenB) vaccine at each injection; 4CMenB 0-6 group, administered 4CMenB at injections 1 and 3, meningococcal serogroups ACWY-CRM glycoconjugate vaccine (MenACWY-CRM) at injection 2; MenACWY group, administered MenACWY-CRM at injection 1, placebo at injection 2, 4CMenB at injection 3.

Severe defined as preventing normal activity or, for erythema, swelling, and induration, diameter >100 mm, or for fever, body temperature ≥40°C.

Number of participants: 885, 823, 765 in 4CMenB 0-2-6 group for injection 1, 2, 3, respectively; 894, 813, 759 in 4CMenB 0-6 group for injection 1, 2, 3, respectively; 178, 161, 148 in MenACWY group for injection 1, 2, 3, respectively.

**Supplementary Table 6.** Number and percentage of participants reporting unsolicited adverse events within 30 days of receiving a vaccine dose and throughout the study period (unsolicited safety set)

|  | **4CMenB 0-2-6 group,  n (%)  N=893** | **4CMenB 0-6 group,  n (%)  N=900** | **MenACWY group,  n (%) N=178** |
| --- | --- | --- | --- |
| **Within 30 days of any vaccination** | | | |
| Unsolicited AEs | 264 (29.6) | 285 (31.7) | 53 (29.8) |
| Related unsolicited AEs | 66 (7.4) | 56 (6.2) | 10 (5.6) |
| Serious AEs | 9 (1.0) | 11 (1.2) | 0 |
| Related serious AEs | 0 | 2 (0.2) | 0 |
| Medically attended unsolicited AEs | 127 (14.2) | 155 (17.2) | 17 (9.6) |
| Unsolicited AE leading to withdrawal | 2 (0.2) | 2 (0.2) | 0 |
| Unsolicited AESI | 1 (0.1) | 1 (0.1) | 0 |
| Deaths | 1 (0.1) | 0 | 0 |
| **Throughout study period** | | | |
| Unsolicited AEs | 349 (39.1) | 384 (42.7) | 69 (38.8) |
| Related unsolicited AEs | 66 (7.4) | 56 (6.2) | 10 (5.6) |
| Serious AEs | 20 (2.2) | 22 (2.4) | 5 (2.8) |
| Related serious AEs | 0 | 2 (0.2) | 0 |
| Medically attended unsolicited AEs | 238 (26.7) | 288 (32.0) | 44 (24.7) |
| Unsolicited AE leading to withdrawal | 6 (0.7) | 4 (0.4) | 1 (0.6) |
| Unsolicited AESI | 1 (0.1) | 1 (0.1) | 0 |
| Deaths | 1 (0.1) | 1 (0.1) | 0 |

AE, adverse event; AESI, adverse event of special interest; N, number of participants in group; n, number of participants in category.

4CMenB 0-2-6 group, three doses of 4-component meningococcal serogroup B (4CMenB) vaccine at study months 0, 2, 6; 4CMenB 0-6 group, two doses of 4CMenB at study months 0, 6; MenACWY group, meningococcal serogroups ACWY-CRM glycoconjugate vaccine at month 0.
